# Common Iliac Artery Thrombosis following Pelvic Surgery Resulting in Kidney Allograft Failure Successfully Treated by Percutaneous Transluminal Angioplasty with Balloon-Expandable Covered Stent

**DOI:** 10.1155/2015/291796

**Published:** 2015-08-18

**Authors:** Maheswara S. Golla, Subasit Acharjee, Bertrand L. Jaber, Lawrence A. Garcia

**Affiliations:** Department of Medicine, St. Elizabeth's Medical Center, Department of Medicine, Tufts University School of Medicine, Boston, MA 02135, USA

## Abstract

We report the case of a 66-year-old woman who developed acute kidney allograft failure due to thrombotic occlusion of the common iliac artery after hysterectomy requiring emergent allograft rescue. She underwent percutaneous transluminal angioplasty with endovascular balloon expandable covered stent graft placement in the right common iliac artery. Although there are a handful of case reports of acute limb ischemia secondary to acute common iliac artery thrombosis, this is the first case reported in the literature resulting in successful kidney allograft rescue following pelvic surgery.

## 1. Background

Arterial thrombosis causing late acute kidney allograft failure is extremely rare. Pelvic or abdominal surgeries may place kidney allografts implanted in the pelvis at risk for injury [1]. In the literature of pelvic surgery complications, injury to the common iliac artery or external iliac artery has been reported, and required either surgical or endovascular repair [2–4]. We report a case that may represent the first in the literature of a kidney allograft rescue following pelvic surgery.

## 2. Case Presentation 

A 66-year-old woman with end-stage renal disease in the setting of type-2 diabetes mellitus, hypertension, kidney stones, and renal artery stenosis had received an unrelated living-donor kidney transplant 7 years earlier. She also had a history of chronic obstructive pulmonary disease, coronary artery disease, heart failure with preserved left ventricular ejection fraction, and atrial fibrillation (on rivaroxaban, an orally active direct factor Xa inhibitor) for which she had undergone atrioventricular nodal ablation and insertion of a permanent pacemaker. She presented with excessive uterine bleeding. The workup demonstrated a pelvic mass and fluid-filled uterus. She underwent an elective hysteroscopy with dilation and curettage, which revealed pyometra. The intraoperative course was complicated by bleeding and uterine perforation requiring total abdominal hysterectomy and bilateral salpingooophorectomy. She lost 300 mL of blood and received intraoperatively 3.2 liters of crystalloids. There was no documented intraoperative hypotension. Pulses were equally palpable in both lower extremities before and after surgery. The patient developed anuria in the immediate postoperative period, and furosemide (40 mg) was administered intravenously with no response. The patient was reintubated for acute respiratory failure, and her anuria persisted.

## 3. Investigations

**Figure 1:**
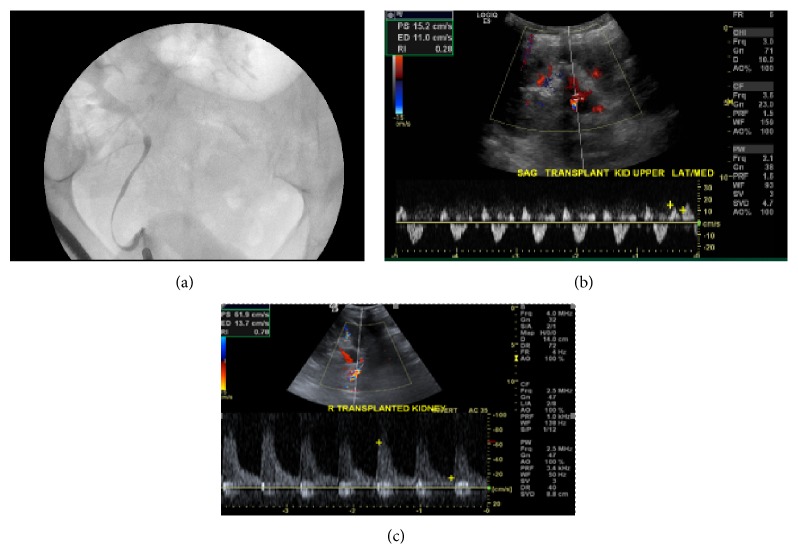
Retrograde pyelography of the kidney allograft showing patency of the ureter, pelvis, and calyces (a). Preprocedural color Doppler of kidney allograft showing renal arterial flow reversal during diastolic phase (b), with normalization of arterial flow pattern on day 1 after the intervention (c).

The urology service was initially consulted, and the patient underwent a cystoscopy with retrograde ureterogram, which revealed normal iodinated contrast filling and caliber of the ureter and intrarenal collective system of the kidney allograft (Figure 1(a)). The nephrology service was consulted approximately 4 hours from the onset of anuria and recommended a Duplex ultrasound of the kidney allograft to assess the renal vasculature. The study demonstrated reversal of flow within multiple renal arterial branches during diastole (Figure 1(b)), which was suspicious for either arterial or venous occlusive disease. Of note, the patient was receiving tacrolimus and vancomycin, and both serum trough levels were therapeutic. The patient was initiated on systemic anticoagulation.

**Figure 2:**
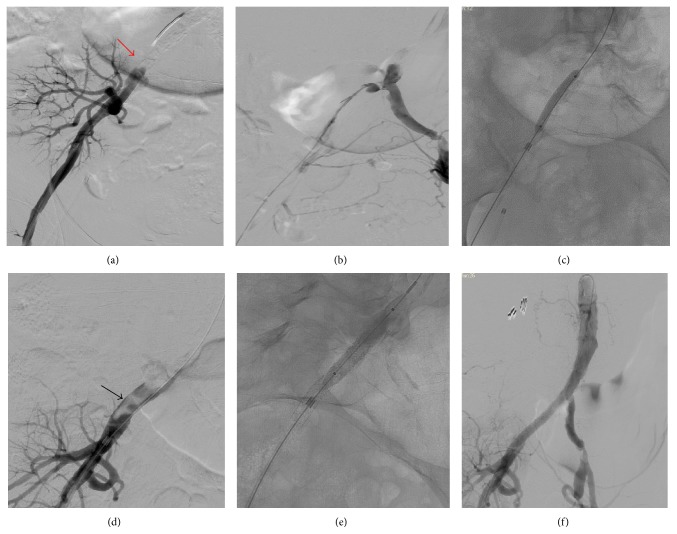
Aortoiliac and femoral angiogram showing complete occlusion (red arrow) of the right common iliac artery proximal to the kidney allograft anastomosis (a). Collaterals from the left iliac and femoral arteries feeding right-sided vessels below the occlusion (b). Balloon angioplasty of the right common iliac artery (c). Partial recanalization and visible floating thrombus (black arrow) of the right common iliac artery after balloon angioplasty (d). Balloon assisted covered stent (iCast) deployment and postdilation of the stent (e). Postintervention angiogram shows normal flow to the kidney allograft (f).

The vascular medicine service was consulted immediately, and emergent iliac and renal angiography was performed within 6 hours from the onset of anuria. Iliac angiography revealed a thrombus obstructing 99% of the proximal right common iliac artery (Figure 2(a)). The length of the arterial segment involved was 50 mm. The anastomosis of the kidney allograft to the right common iliac artery was patent and free of disease. There was no evidence of iliac vein trauma or disruption to account for the anuria.

## 4. Treatment

We performed the intervention through retrograde right common femoral artery in an intraluminal position. The culprit lesion was located at the origin of the right common iliac artery, which was 50 mm in length. The lesion was crossed intraluminally by a 0.035′′ Terumo guide wire. A 5 × 40 mm Admiral Xtreme (Medtronic, Inc.) balloon was advanced over the guide wire on to the lesion and predilated by two inflations with a maximum pressure of 14 atmospheres (Figures 2(c) and 2(d)). A 7 × 59 mm iCast covered stent (Atrium Medical Corporation, Hudson, NH), a balloon-expandable stainless steel encapsulated stent, was then deployed in the right common iliac artery. To control sandwiched thrombi (thrombi in between the vessel wall and covered stent) prolapse from the edge of the stent to the intraluminal area and to cover the edge of the culprit lesion completely, we deployed a second 7 × 22 mm iCast stent more distally (Figure 2(e)). Both stents were postdilated with a 8 × 40 mm Dorado PTA balloon (Bard Peripheral Vascular, Inc., Tempe, AZ) (Figure 2(e)) to minimize in-stent restenosis and stent thrombosis. There was no residual stenosis and the pressure gradient dropped to zero with normal flow noted through the iliac artery and the kidney allograft artery (Figure 2(f)). We used a total of 80 mL of Iohexol (Omnipaque, GE Healthcare) due to the unavailability of carbon dioxide for the angiogram during the intervention.

## 5. Outcome and Follow-Up

**Figure 3:**
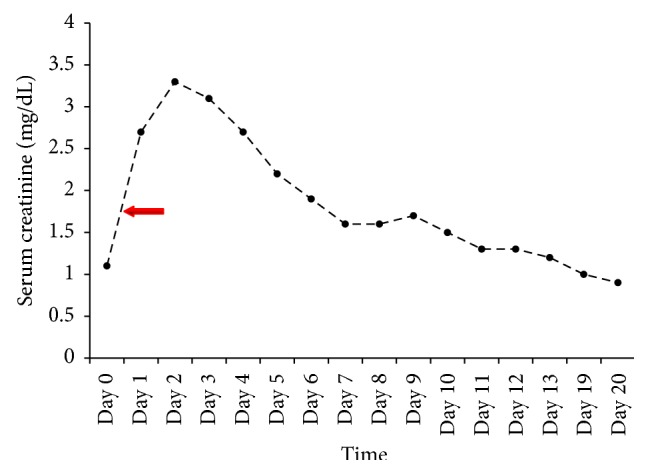
Time course of the serum creatinine in relation to the pelvic surgery (day 0) and the endovascular intervention (red arrow: time of intervention).

Following the procedure, the anuria began to resolve within hours and corrected overnight. The serum creatinine level, which had peaked at 3.3 mg/dL, declined and returned to the baseline value of 0.9 mg/dL over two weeks (Figure 3).

One week after the intervention, a repeat Duplex ultrasound of the right common iliac artery revealed normal flow and patent stents (Figure 1(c)). There was normal Doppler flow to the kidney allograft. The patient remained asymptomatic and her serum creatinine obtained at 8 months of follow-up was 1.0 mg/dL.

## 6. Discussion

Late kidney allograft failure after pelvic surgery is extremely rare. To our knowledge, this is the first case to report the successful rescue of a kidney allograft after pelvic surgery by endovascular intervention. One similar case was reported in 1990, although the kidney allograft was completely thrombosed and infarcted [1]. A handful of iliac artery injury cases have been reported following pelvic surgeries [2–4]. The etiology of vascular complications can be classified as thrombotic and embolic and from other causes (external or internal compression or injury). Thrombosis may arise as a complication of intraoperative manipulation of vital structures [1–4]. In our case, Doppler arterial scan of the allograft renal artery showed “flow reversal in diastole,” which may be a consequence of either arterial obstruction or venous obstruction/occlusion similar to a phlegmatic limb (Figure 1(a)). The angiogram revealed right common iliac artery subtotal occlusion with evident thrombus (Figure 2(a)) but a patent renal allograft anastomosis. Hence, this late acute allograft failure was attributed to decreased perfusion pressure to the kidney allograft secondary to acute arterial inflow obstruction. Prompt intervention within the 6 hours after initial anuria successfully rescued the renal allograft.

The Covered versus Balloon-Expandable Stent Trial (COBEST), a multicenter, randomized, controlled trial involving iliac arteries in patients with severe aortoiliac occlusive disease, showed that covered stents had a significantly better primary patency for Trans-Atlantic Inter-Society Consensus (TASC) C and D lesions compared with the noncovered stents [5]. Among the balloon-expandable covered stents, iCast stents are approved in the US for aortoiliac occlusions or stenotic disease. In our case, the patient underwent balloon angioplasty with iCast stenting to the right common iliac artery. The patient remains symptom-free 8 months after the intervention, with an excellent kidney allograft function.
